# Impact of CytoSorb® on interleukin-6 in cardiac surgery

**DOI:** 10.3389/fcvm.2023.1166093

**Published:** 2023-08-30

**Authors:** Daniela Geisler, Noemi Arleth, Johannes Grabenwöger, Zsuzsanna Arnold, Thomas Aschacher, Bernhard Winkler, Markus Mach, Martin Grabenwöger

**Affiliations:** ^1^ Department of Cardiovascular Surgery, Clinic Floridsdorf, Vienna, Austria; ^2^ Institute of Cardiovascular Research, Karl Landsteiner Society, Vienna, Austria; ^3^ Medical Faculty, Sigmund Freud Private University, Vienna, Austria; ^4^ Department of Cardiac Surgery, Medical University of Vienna, Vienna, Austria

**Keywords:** IL-6, cytokine storm, CytoSorb®, hemadsorption, cardiac surgery

## Abstract

**Objective:**

Cardiac surgery is known to activate a cascade of inflammatory mediators leading to a systemic inflammatory response. Hemadsorption (HA) devices such as CytoSorb® have been postulated to mitigate an overshooting immune response, which is associated with increased morbidity and mortality, and thus improve outcome. We aimed to investigate the effect of CytoSorb® on interleukin (IL)-6 levels in patients undergoing complex cardiac surgery in comparison to a control group.

**Methods:**

A total of 56 patients (28 CytoSorb®, 28 control) undergoing acute and elective cardiac surgery between January 2020 and February 2021 at the Department of Cardiac and Vascular Surgery, Clinic Floridsdorf, Vienna, were retrospectively analyzed. The primary endpoint was the difference in IL-6 levels between the CytoSorb® and control group. Secondary endpoint was periprocedural mortality.

**Results:**

CytoSorb®, installed in the bypass circuit, had no significant effect on IL-6 levels. IL-6 peaked on the first postoperative day (HA: 775.3 ± 838.4 vs. control: 855.5 ± 1,052.9 pg/ml, *p* = 0.856). In total, three patients died in the HA group, none in the control (logistic regression model, *p* = 0.996). Patients with an increased Euroscore II of 7 or more showed a reduced IL-6 response compared to patients with an Euroscore II below 7 (178.3 ± 63.1 pg/ml vs. 908.6 ± 972.6 pg/ml, *p*-value = 0.00306).

**Conclusions:**

No significant reduction of IL-6 levels or periprocedural mortality through intraoperative HA with CytoSorb® in patients undergoing cardiac surgery was observed. However, this study was able to show a reduced immunologic response in patients with a high Euroscore II. The routine application of CytoSorb® in cardiac surgery to reduce inflammatory mediators has to be scrutinized in future prospective randomized studies.

## Introduction

Cardiac surgery evokes an unpredictable activation of the complement cascade and stimulation of the immune system induced by surgical trauma, cardiopulmonary bypass (CPB) through sheer stress, artificial surfaces and reperfusion injury. A normal immune response results in a controlled inflammation process involving pro- and anti-inflammatory cytokines. In case of a dysregulation, inflammatory mediators are excessively released, which is referred to as “cytokine storm” (1, 2). This hyperactivation may result in a systemic inflammatory response syndrome (SIRS) and consequently septic shock. Cytokine-induced vasodilatation and increased capillary permeability cause hemodynamic depression and organ dysfunction, linked to increased morbidity and mortality (3–5). Interleukin (IL-) 6 plays a crucial role as early indicator of inflammation prior to C-reactive protein (CRP) and is therefore routinely used in the intensive care setting (6). IL-6 is induced by tumor necrosis factor in response to severe injury and infection and stimulates the synthesis of acute-phase-proteins such as CRP in the liver. Elevated IL-6 levels were not only shown to correlate with the severity of sepsis but also to be highly predictive of adverse outcome following cardiac surgery (1, 7, 8). Bauer et al. also found IL-6 to be predictive for prolonged mechanical ventilation and thus longer stay at the ICU (7).

Hemadsorption (HA) devices have been postulated to reduce excess cytokine levels—as produced in a cytokine storm—and thus attenuate an overshooting immune response and ultimately prevent multi-organ failure (9). The CytoSorb® adsorber (CytoSorbents Europe GmbH, Berlin, Germany) is the most widely-used cytokine filter and consists of a crosslinked divinyl benzol-polymer filtering small and mid-size hydrophobic molecules up to a size of 60 kDa. It was designed for the removal of inflammatory mediators in SIRS, sepsis and septic shock and is now increasingly adopted in cardiac surgery to mitigate the inflammatory response induced by CPB (10, 11). Reported results are inconsistent and in the scarce literature of HA in cardiac surgery the effect of CytoSorb® on inflammatory cytokines remains questionable (10–13). The aim of this retrospective study was to investigate whether the use of CytoSorb® during CPB in patients undergoing cardiac surgery has an effect on IL-6 levels and secondarily on periprocedural mortality. Furthermore, possible factors leading to increased IL-6 were analyzed and reported out of concurrence.

## Materials and methods

### Study design and patients

The study protocol was reviewed and approved by the ethics committee of Vienna, reference number EK 21-039-VK. Written informed consent for participation was not required for this study in accordance with the national legislation and the institutional requirements.

#### Study cohort

A total of 56 patients who underwent elective or acute cardiac surgery with (*n* = 28) and without (=28) CytoSorb® between January 2020 and February 2021 at the Department of Cardio-Vascular Surgery Vienna, Clinic Floridsdorf, Vienna, Austria, were retrospectively analyzed. CytoSorb® was employed non-randomly at the surgeon's discretion, predominantly in endocarditis, redo- and high-risk surgeries. A control group was chosen within the same time period.

#### Inclusion criteria

Criteria for inclusion for both groups were: coronary artery bypass surgery, aortic-, mitral- and/or tricuspid valve repair/replacement, surgery of the ascending aorta and aortic arch including surgeries with circulatory arrest or combined procedures and serum levels of IL-6 available at baseline and in the postoperative period.

#### Hemadsorption protocol

According to the manufacturer's recommendation the CytoSorb® 300 ml adsorber had been installed in the CPB circuit (Stöckert S5 LivaNova, USA, Inc., Arvada, CO.) with a side arm coming from the venous outflow tube and given back to the venous reservoir prior to the oxygenator. Blood was pumped actively through the CytoSorb® cartridge with a standardized rate of 200 ml/min by a roller pump. The CPB circuit was primed according to institutional standards (1,700 ml Elomel saline solution + 10.000 IE heparin). In this study, CytoSorb® filtering was active only during CPB time.

IL-6 is routinely assessed in laboratory measurements at the intensive care unit (ICU) at our department to monitor the postoperative course of inflammation, as elevated IL-6 levels were shown to be predictive of the course in the ICU following cardiac surgery. In general, the first measurement postoperatively is about 6 h after the operation, then routine laboratory measurements are around 6 a.m. Thus, time points can be described as follows: before surgery, 6 h after end of surgery, first postoperative (POD 1) and second postoperative day (POD 2). For the quantification of IL-6 electrochemiluminescence sandwich immunoassay ECLIA (Roche Diagnostics) was used.

### Endpoints

Primary endpoint was difference in IL-6 levels at its peak, which was on the first POD, between the HA and control group. Secondary endpoint was periprocedural mortality, defined as death occurring ≤30 days after the index procedure, >30 days but during the index hospitalization. Additionally, clinical parameters, duration of surgery, aortic cross-clamp and CPB time, catecholamine use, ICU and overall hospital stay as well as relevant laboratory parameters such as leucocytes count, c-reactive protein (CRP) were assessed.

#### Sample size calculation

Sample size calculations were performed for the primary outcome. A strong effect of CytoSorb® on IL-6 levels was assumed (effect size *d* = 0.8), at a level of significance of *α* = 5% and a power of 80%. The number of patients per group was calculated to be 21. We estimated a drop-out quote of one third due to missing data, therefore an additional of 7 patients were included.

#### Statistical analysis

Patient and perioperative data was collected from the electronic hospital records. Patient records were pseudoanonymized for further processing. Statistical analysis was performed using the open-source statistical software package R [version 4.1.0, 2021-05-18, R Core Team (2021). R: A language and environment for statistical computing. R Foundation for Statistical Computing, Vienna, Austria. https://www.R-project.org].

The *primary endpoint* IL-6 serum levels [in pg/ml] on the first POD (IL-6 POD 1) were compared in a Wilcoxon sum rank test, as IL-6 was not normally distributed. Comparison of IL-6 levels at all timepoints were additionally reported using Wilcoxon signed rank tests and *p*-values were Bonferroni corrected. Nevertheless, the primary endpoint remained IL-6 POD 1 levels and these outcomes were reported out of concurrence.

For the *secondary endpoint* periproceural mortality, a logistic regression model was used.

As patients who had Cytosorb installed in the CPB are generally sicker, this patient cohort represents a real-world setting. In c*onfounder analyses* the influence of significantly different baseline parameters (ES II, number of redo surgeries, ascending aortic replacements, aortic arch replacements, surgery time, and cardiopulmonary bypass time) on peak IL-6 levels were assessed. In case of ratio scaled parameters linear regression models were used to estimate the logarithm of IL-6. The logarithm was used to reduce the right-skewness of IL-6. In case of dichotomous baseline parameters, a Wilcoxon sum rank test was used. Additionally, a propensity score matching was performed matching age, Euroscore II, CPB time, surgical duration, see Supplementary Tables S1–S4, which contain all statistics of Tables 1–4 based on the propensity score matched data set.

**Table 1:** Interval and ratio scaled baseline parameters of CytoSorb® and control group.

Time	Parameter	HA (*n* = 28)				Control (*n* = 28)				*t*-Test		
		Mean	SD	Min	Max	Mean	SD	Min	Max	*t*	Dof	*p*
Preop	Age	62.8	14.7	32.0	82.0	67.3	14.5	24.0	81.0	−1.160	53.98738	0.251
	BMI	29.4	14.7	18.7	101.0	26.8	4.1	20.5	35.2	0.904	31.27468	0.373
	Euroscore II	5.4	6.0	0.7	23.7	2.5	2.2	0.5	9.8	2.395	34.07519	**0**.**022**
	Heart rate in bpm	73.5	10.1	54.0	90.0	74.0	11.8	53.0	95.0	−0.183	52.75003	0.856
	Respiratory rate in bpm	11.8	1.0	9.0	14.0	11.9	0.6	10.0	13.0	−0.806	44.27324	0.425
	Body temperature in °C	37.1	0.4	36.0	37.7	37.0	0.4	36.0	37.5	0.710	53.74381	0.481
Baseline	Hemoglobin (g/dl)	13.3	1.7	11.2	18.0	13.6	1.5	10.5	16.8	−0.713	52.97789	0.479
	Leukocyte count (G/L)	7.3	2.2	3.0	12.4	7.6	2.4	3.9	13.3	−0.449	53.77825	0.655
	Albumin (g/dl)	42.5	2.2	37.0	48.0	41.6	5.0	29.0	49.0	0.799	37.36149	0.429
	Bilirubin (mg/dl)	0.6	0.3	0.2	1.4	0.6	0.4	0.2	2.4	−0.529	50.49803	0.599
	CRP (mg/L)	9.0	25.3	0.3	135.8	6.0	15.8	0.3	83.2	0.522	45.14523	0.604
	IL6 (pg/ml)	16.6	39.5	1.5	206.0	7.8	12.5	1.5	67.7	1.124	32.33027	0.269
	Procalcitonin (ng/ml)	0.0	0.0	0.0	0.1	0.0	0.1	0.0	0.3	−1.311	30.04276	0.200
Intraop	Surgery duration (min.)	300.7	72.0	168.0	454.0	260.1	63.7	183.0	422.0	2.231	53.21981	**0**.**030**
	CPB (min.)	155.7	50.2	81.0	253.0	125.2	41.5	56.0	239.0	2.472	52.18499	**0**.**017**
	ACC (min.)	93.3	32.7	0.0	155.0	86.1	38.1	27.0	202.0	0.764	52.80028	0.448

Bold parameters indicate a significant difference between both groups.

BMI, body mass index; Dof, degrees of freedom; Max, maximum; Min, minimum; SD, standard deviation, *p*, *p*-value. ACC, aortic cross clamp, BMI, body mass index; CPB, cardiopulmonary bypass; CRP, c-reactive protein; HA, hemadsorption; IL6, interleukin 6.

**Table 2:** Dichotomous distributed baseline parameters of CytoSorb® and control group.

Time	Parameters	HA (*n* = 28)	Control (*n* = 28)	*χ*^2^ test	
				*χ* ^2^	*p*-value
Preop	Sex (male)	15 (53.6)	21 (75.0)	1.944	0.163
	Art. Hypertension	21 (75.0)	21 (75.0)	0.000	1.000
	Pulmonary hypertension	2 (7.1)	4 (14.3)	0.187	0.666
	Hyperlipidemia	20 (71.4)	19 (67.9)	0.000	1.000
	COPD	5 (17.9)	5 (17.9)	0.000	1.000
	Diabetes mellitus	7 (25.0)	9 (32.1)	0.350	0.774
	Peripheral artery disease	0 (0)	0 (0)	NA	NA
	Cerebrovascular disease	7 (25.0)	5 (17.9)	0.106	0.745
	Marfan syndrome	3 (10.7)	0 (0)	1.409	0.234
Indication	Aortic stenosis	7 (25.0)	9 (32.1)	0.088	0.767
	Aortic regurgitation	5 (17.9)	2 (7.1)	0.653	0.419
	Combined aortic vitium	0 (0)	1 (3.6)	0.000	1.000
	Mitral regurgitation	5 (17.9)	8 (28.6)	0.401	0.527
	Mitral stenosis	1 (3.6)	1 (3.6)	0.000	1.000
	Tricuspid regurgitation	2 (7.1)	4 (21.4)	0.187	0.666
	Aneurysm	12 (42.9)	3 (10.7)	5.828	**0**.**016**
	Dissection	5 (17.9)	0 (0)	3.514	0.060
	Coronary artery disease	7 (25.0)	15 (53.6)	3.668	0.055
	Myocardial infarction	0 (0)	2 (7.1)	0.519	0.471
Surgery	Re-do surgery	12 (42.9)	0 (0)	12.833	**0**.**000**
	Combined surgery	19 (67.9)	13 (46.4)	1.823	0.177
	AVR mechanical	2 (7.1)	1 (3.6)	0.000	1.000
	AVR biological	8 (28.6)	10 (35.7)	0.082	0.775
	Mitral valve replacement	5 (17.9)	4 (14.3)	0.000	1.000
	Mitral valve repair	1 (3.6)	5 (17.9)	1.680	0.195
	Tricuspid valve repair	2 (7.1)	4 (14.3)	0.187	0.666
	CABG	8 (28.6)	15 (53.6)	2.656	0.103
	Ascending aorta replacement	11 (39.9)	3 (10.7)	7.547	**0**.**023**
	Aortic arch replacement (partial/full)	10 (35.7)	1 (3.6)	7.240	**0**.**007**
	Bentall procedure	1 (3.6)	1 (3.6)	0.000	1.000
	ECMO	1 (3.6)	2 (7.1)	0.000	1.000

Bold parameters indicate a significant difference between both groups. Columns 3 and 4 show absolute numbers (*n*=) and percentages in brackets (%).

AVR, aortic valve replacement; COPD, chronic obstructive pulmonary disease; CABG, coronary artery bypass grafting; ECMO, extracorporeal membrane oxygenation; HA, Hemadsorption.

**Table 3:** Inflammatory blood parameters of both groups specified by median (1st quartile, 3rd quartile).

		HA (*n* = 28)	Control (*n* = 28)	*p*-value
IL6 [pg/ml]	Baseline	3.9 (2.4, 9.8)	4.0 (2.8, 7.3)	0.974
	6 h postop	357.5 (261.0, 667.8)	479.5 (238.0, 883.8)	0.533
	POD1	398.5 (215.8, 918.8)	392 (245.3, 1,073.3)	0.856
	POD2	203.0 (133.5, 458.5)	253.0 (91.0, 323.3)	0.720
CRP [mg/L]	Baseline	2.1 (0.9, 6. 5)	1.0 (0.5, 4.9)	0.359
	6 h postop	14.9 (8.2, 18.7)	11.41 (8.9, 14.8)	0.599
	POD1	67.3 (48.9, 90.8)	73.1 (56.1, 89.5)	0.890
	POD2	232.5 (157.7, 275.4)	214.0 (186.4, 273.7)	0.955
Leukocyte count (G/L)	Baseline	7.2 (5.8, 7.9)	7 (5.9, 9.2)	0.831
	POD1	11.0 (7.7, 15.1)	12.2 (9.9, 15.2)	0.298
	POD2	12.2 (8.8, 17.2)	11.2 (9.3, 15.1)	0.699

The *p*-value refers to the Wilcoxon rank sum test.

CRP, c-reactive protein; HA, hemadsorption; IL6, interleukin 6; POD, postoperative.

**Table 4:** Postoperative outcome parameters.

	HA (*n* = 28)	Control (*n* = 28)	*χ*^2^^a^/W^b^	*p*-value
Periprocedural mortality, *n* (%)	3 (10.7)	0 (0)	0.011^a^	0.996
ICU stay, in days median ± mad	5.0 ± 2.2	5.0 ± 2.2	374^b^	0.771
Atrial fibrillation *de novo*, *n* (%)	4 (14.3)	6 (21.4)	0.0004^a^	0.984
Stroke rate, *n* (%)	1 (3.6)	1 (3.6)	0^a^	1.000
Ventilation time median ± mad	17.5 ± 17.0	9.0 ± 7.4	344^b^	0.431
Reintubation, *n* (%)	4 (14.3)	0 (0)	0.043^a^	0.836
Acute kidney injury, *n* (%)	4^c^ (14.3)	7^c^ (25)	0.452^a^	0.501
Renal replacement therapy, *n* (%)	0 (0)	0 (0)	0.000^a^	1.000

^a^ Pearson's *χ*^2^ test.

^b^ Wilcoxon rank sum test with continuity correction, mad … median deviation of the median.

^c^ Patients with acute kidney injury (AKI) stage 1: increase in serum creatinine by 0.3 mg/dl or more within 48 h or increase in serum creatinine to 1.5 times baseline according to KDIGO-criteria.

In *exploratory analyses* we investigated possible factors (impact of oxygenators, steroid application) affecting IL-6 levels, since those were high in comparison to other studies. Three different oxygenators were used: Terumo Capiox® FX25 Advance Oxygenator, Eurosets Horizon and Getinge Quadrox-i®. Additionally, the effect of HA on CRP serum levels, leukocyte count, norepinephrine levels was tested in Wilcoxon rank sum tests. Postoperative outcome parameters including ICU stay, atrial fibrillation *de novo*, stroke rate, ventilation time and reintubation were evaluated. The length of the ICU stay was tested in a Poisson regression model. To evaluate the validity of the study sample, the prognostic effect on 30-day mortality of known risk factors such as ES II, troponin I, and lactate levels on POD 1 were analyzed in a ROC analysis and opposed to peak IL-6. The area under curve (AUC), the sensitivity, and the specificity were reported.

## Results

In this retrospective study, 56 patients who underwent cardiac surgery for different indications were included: 28 patients with CytoSorb® installed in the bypass circuit and 28 patients in the control group, with no HA device. The groups were comparable in terms of age and sex (see Tables 1, 2). Following baseline parameters showed to be significantly different between both groups: ES II, number of redo surgeries, ascending aortic replacements, aortic arch replacements, and cardiopulmonary bypass time (CPB, see Tables 1, 2). Baseline parameters are depicted in Table 1 (containing the normal distributed parameters) and Table 2 (containing the dichotomous parameters).

### Primary endpoint: impact of CytoSorb® on IL-6 levels

**Figure 1:**
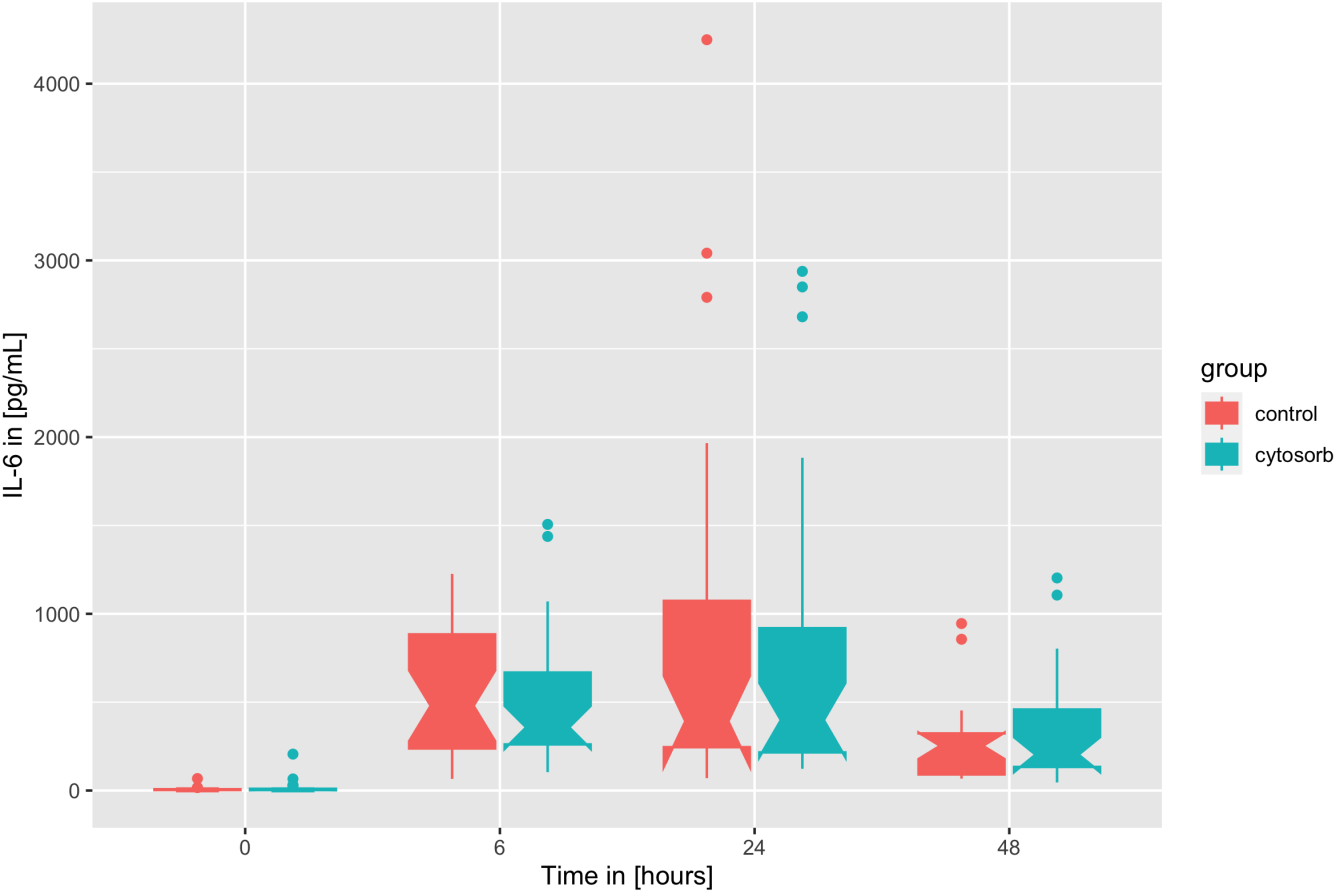
Timely course of IL-6 as boxplots over time at baseline, 6 h after surgery, on the first postoperative day (POD 1), on the second postoperative day (POD 2).

Primary endpoint results are depicted in Table 3 and Figure 1. Preoperative IL-6 levels were 16.6 ± 39.5 pg/ml in the CytoSorb® vs. 7.8 ± 12.5 pg/ml in the control group. Six hours after surgery the IL-6 levels showed a strong increase (HA: 512.6 ± 391.2 vs. control: 554.1 ± 340.8 pg/ml) and peaked on the first POD (HA: 775.3 ± 838.4 vs. control: 855.5 ± 1,052.9 pg/ml, not significant). The IL-6 POD 1 levels showed no significant difference in a Wilcoxon sum rank test (*p* = 0.856, see Table 3)*.* Two days after surgery the IL-6 levels halved (IL-6 POD 2, HA: 347.6 ± 310.8 vs. control: 283.5 ± 225.9 pg/ml). *At all times CytoSorb® did not exhibit a significant effect on IL-6 levels when compared to the control group*. All significantly different baseline parameters were investigated in confounder analyses (see “Confounder analyses” at the end of the “Results” section). In the propensity score matched data set, IL-6 did not show to be significantly different between groups (Supplementary Table S3). Previously significant baseline parameters such as Euroscore II, surgical duration, and CPB time were not significantly different following propensity score matching (Supplementary Table S1).

### Secondary endpoint: impact of CytoSorb® on periprocedural mortality

Although all of three deceased patients were in the HA group, no significant effect was shown in the logistic regression model (OR = *e*^−18.4^, 95%- confidence interval = 0.0–437.5, *p* = 0.996). The three deceased patients were female with ages of 62, 76, and 77 showing high ES II values of 11.3, 14.2, and 19.6, respectively. Neither IL-6 levels of the first POD were indicative (206, 154, 219 pg/ml), nor body temperature (37.4, 37.1, 36.9°C). Serum levels of troponin I on the first POD were 11,563, 1,149, 3,611 µg/L, lactate levels were 3.3, 5.71, 3.22 mmol/L. Indications for surgery were acute aortic dissection, re-aortic valve stenosis and aortic aneurysm, respectively. Surgeries were replacement of the ascending aorta and hemiarch in mild hypothermic circulatory arrest, redo aortic valve replacement and coronary artery bypass grafting, and reoperation with replacement of the ascending aorta and hemiarch in mild hypothermic circulatory arrest. The 62-year old patient died due to liver failure resulting in multiorgan failure on the 4th postoperative day, the 76-year old patient died on the first postoperative day due to right ventricular failure after revision due to hemothorax, and the 77-year old patient died on the 20nd postoperative day due to multiple strokes, bilateral pneumonia requiring veno-venous extracorporeal membrane oxygenation and consequently followed by SIRS.

### Confounder analysis

The impact of the significantly different baseline parameters on IL-6 levels on the first POD (i.e., IL-6 POD 1 = peak of IL-6) were further analyzed, i.e., ES II, number of redo surgeries, ascending aortic replacements, aortic arch replacements, surgery time, and CPB time.

**Figure 2:**
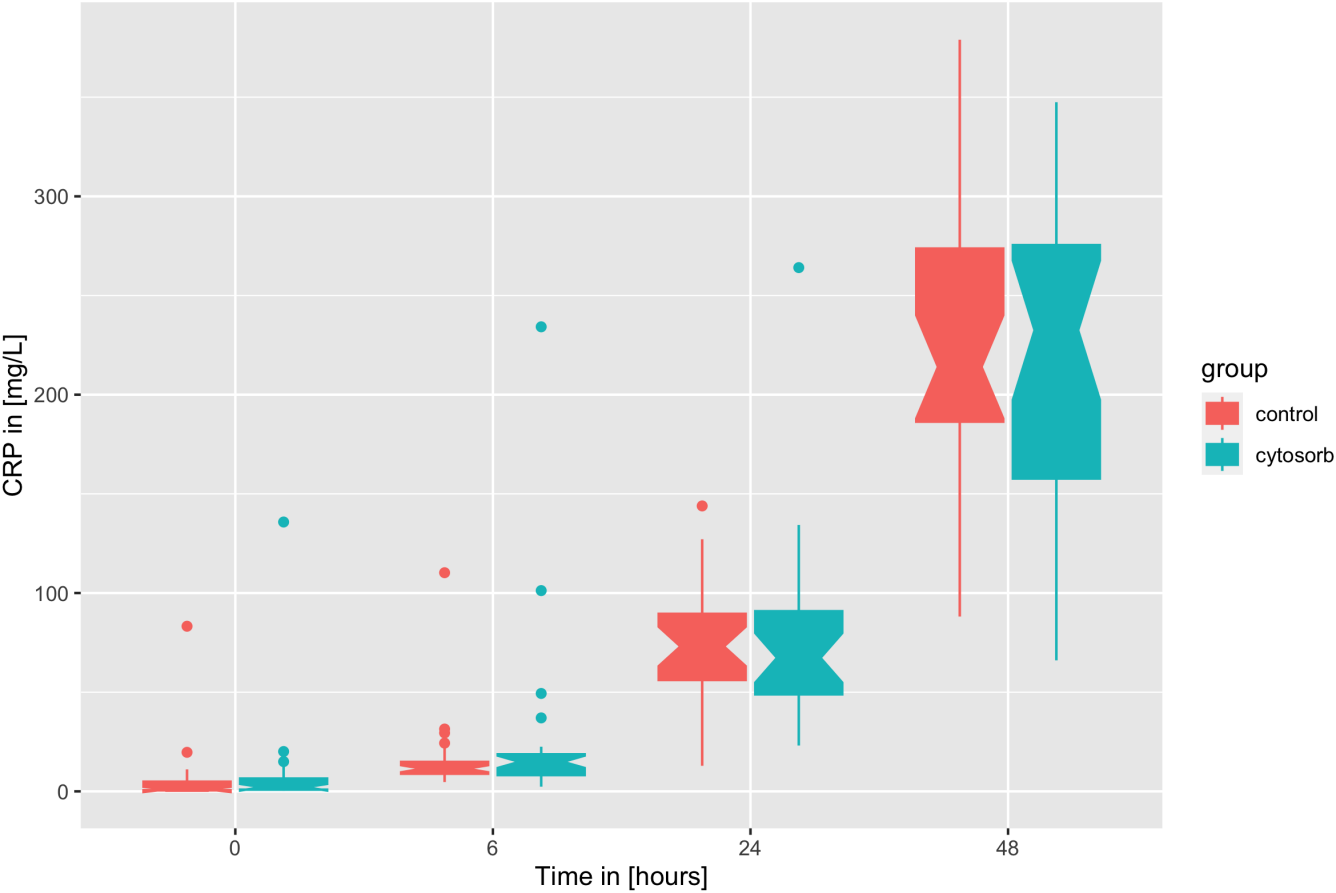
Timely course of CRP as boxplots over time at baseline, six hours after surgery, on the first postoperative day (POD 1), and on the second postoperative day (POD 2).

*ES II* showed a significant effect on IL-6 POD 1 levels [linear regression model: log(IL-6 POD 1) = 6.4–0.069 × ES II, Wald's test *p* (ES II) = 0.023]. Moreover, patient with an increased ES II of 7 or more showed significantly reduced IL-6 POD 1 response (ES II > 7: 178.3 ± 63.1 pg/ml vs. IL-6 levels in ES II < 7: 908.6 ± 972.6 pg/ml, Wilcoxon rank sum test: *W* = 280, *p*-value = 0.003, see Figure 2). Among the significantly different intraoperative parameters, the surgery duration did not show a significant effect on IL-6 POD 1 levels [linear regression model: log(IL-6) = 6.15–0.00002 × surgery duration, *p*(surgery duration) = 0.993], similarly to CPB time [linear regression model: log(IL-6) = 6.19–0.0003 × CPB, *p*(CPB) = 0.923].

IL-6 POD 1 levels were equally high for redo surgery patients vs. in all other patients (439.8 ± 390 pg/dl vs. 559.1 ± 357.9 pg/dl, Wilcoxon rank sum test with continuity correction, *W* = 324.5, *p*-value = 0.1341).

Neither ascending aorta replacement (replacement group: 396.4 ± 319.6 vs. 585.0 ± 371.5 pg/dl in the others, Wilcoxon rank sum test with continuity correction, *W* = 358.5, *p*-value = 0.2059), nor aortic arch replacement did affect the IL-6 POD 1 levels, in case of IL-6 POD 1 was 402.5 ± 376.6 in the group with arch replacement vs. 565.9 ± 358.8 in all others (Wilcoxon rank sum test with continuity correction, *W* = 318.5, *p*-value = 0.08003).

### Exploratory analysis: laboratory parameters and clinical course

Three different *oxygenators* were used in the CPB circuit: horizon, fx25 m, and quadrox-i. There was no significant effect of the different oxygenators on IL-6 POD 1 [horizon: 659.5 ± 755.9 pg/dl, fx25: 825.4 ± 1,048.5 pg/dl, quadrox-i: 938.8 ± 941.1 pg/dl, linear regression model estimating log(IL-6 POD 1) = 6.0 + 0.13 × fx25 (yes or no) + 0.37 × quadrox (yes or no) with horizon as reference, *p*(fx25) = 0.721, *p*(quadrox) = 0.356]. Intraoperative single shot administration of *steroids* did not decrease IL-6 POD 1 [linear regression model: log(IL-6 POD 1) = 6.1 + 0.09 × steroids (yes or no), *p*(steroids) = 0.786]. At the end of the surgery *norepinephrine* dosage was comparable in both groups, i.e., 5.2 ± 4.0 ml/h in the CytoSorb® group and 4.8 ± 6.6 in the control group (Wilcoxon rank sum test with continuity correction, *W* = 336, *p*-value = 0.624).

**Figure 3:**
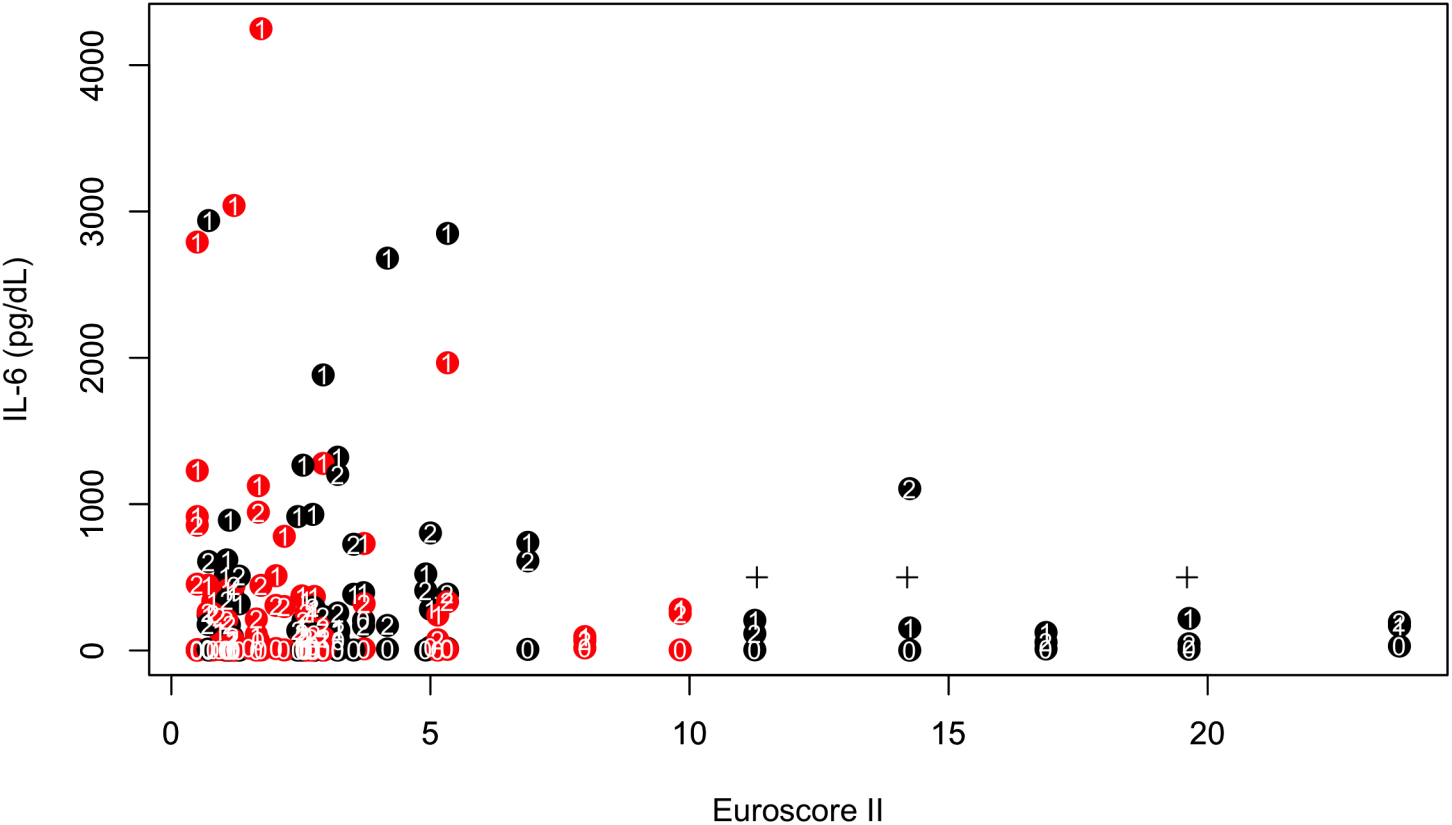
IL-6 at three different timepoints (indicated as white letters: 0 baseline, 1 first postoperative day, 2 second postoperative day) vs. Euroscore II. Red refers to HA, black to control. The cross symbols mark the three deceased patients.

**Figure 4:**
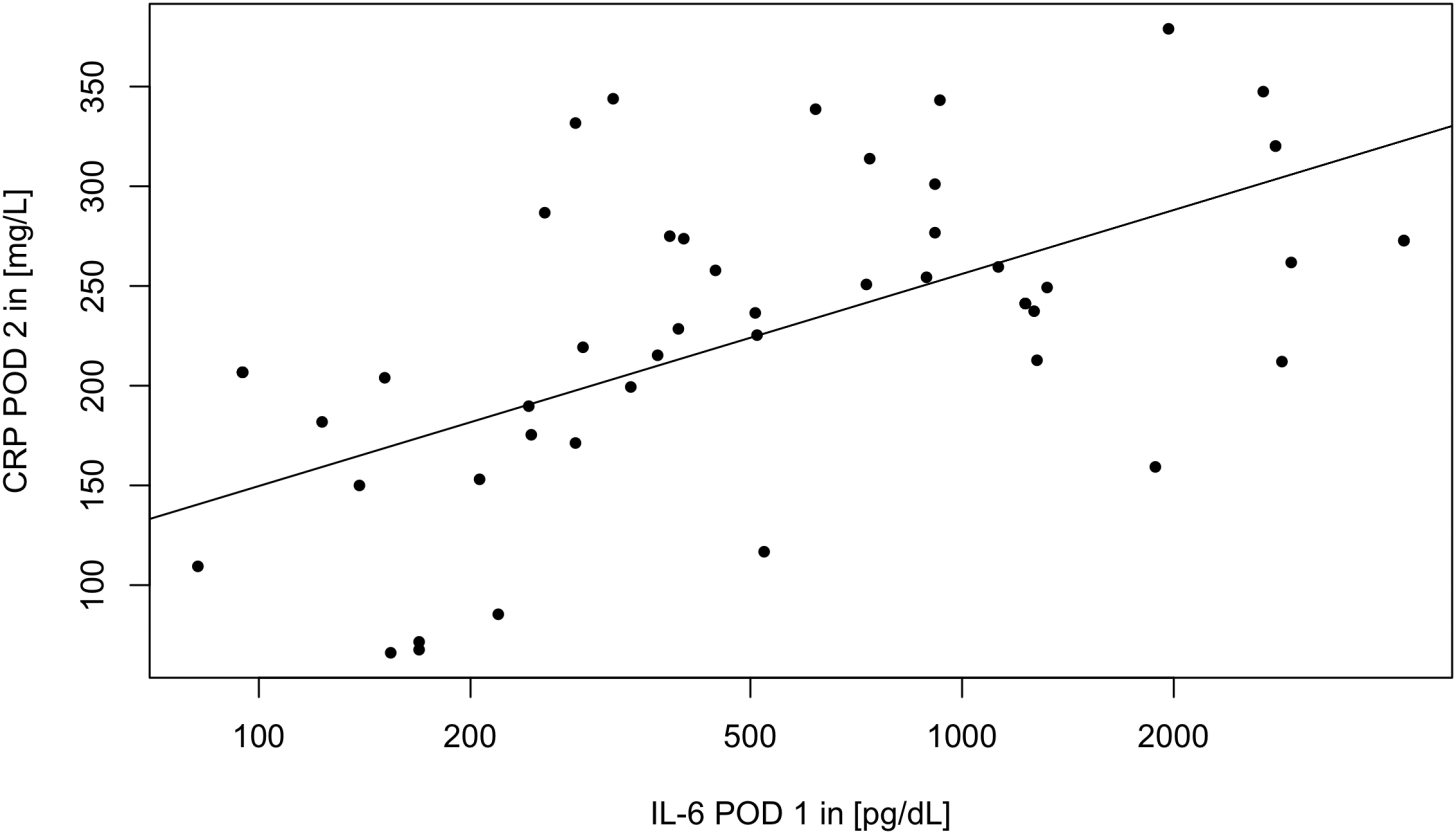
Scatterplot of CRP POD 2 vs. IL-6 POD 1. A regression line was plotted.

Similarly to IL-6, *CRP* serum levels are driven by the surgery but in contrast to IL-6 with a known temporal lag of 24 h (see Figure 3). Again, no effect of CytoSorb® on CRP and leukocyte count was found (see Table 3). The peak CRP levels on the second POD are correlated to the peaking logarithm of IL-6 levels of the first POD [linear regression model: CRP POD 2 = −63.2 + log(IL-6 POD 1), adjusted *R*^2 ^= 0.37, Wald's test *p* < 0.001, see Figure 4].

The ICU stay was 6.6 ± 5.6 days in the CytoSorb® group and 5.3 ± 2.6 in the control group. A Poisson regression model of ICU stay did not significantly differ between both groups (Poisson regression model: *e*^1.7 + 0.2 × Cytosorb^, Wald's test *p* = 0.053).

A powerful prognostic effect of ES II on periprocedural mortality was shown (ROC analysis: AUC = 0.97, Threshold = 10.54, Sensitivity = 100%, Specificity = 96.2%) of troponin I POD 1 (ROC analysis: AUC = 0.93, threshold = 1,137, Sensitivity = 100%, Specificity = 82.7%) and of lactate levels POD 1 (ROC analysis: AUC = 0.96, threshold = 3.105, Sensitivity = 100%, Specificity = 92.5%) but not of IL-6 POD1 (ROC analysis: AUC = 0.79, Threshold = 230.5, Sensitivity = 100%, Specificity = 78.4%).

Postoperative outcome parameters were compared using Pearson's *χ*^2^ test in case of count data and Wilcoxon rank sum test for metric data, respectively, and were not significantly different between groups (see Table 4).

## Discussion

### The effect of intraoperative application of CytoSorb® on IL-6

CytoSorb® applied during CPB in patients undergoing cardiac surgery showed to have no effect on postoperative IL-6 levels in this retrospective single center cohort. Furthermore, no difference in mortality between the CytoSorb® and the control group was observed, i.e., the most relevant clinical endpoint.

CytoSorb® therapy is approved for the non-selective removal of excessive levels of cytokines. To date, there are seven randomized controlled trials (RCT) investigating this role of CytoSorb® applied in cardiac surgery (10–16). Comparing these RCTs, IL-6 levels showed a heterogenous course with peak values ranging from immediately after surgery (12, 16), on admission at ICU (14), 2 h after surgery (11), and 6 h after surgery (10, 13). The fact that the half-life of IL-6 lies within minutes, suggests that once the causative trigger is eliminated, IL-6 levels should decrease rapidly, i.e., after surgery, remaining elevated only in cases of prolonged immune answer e.g., sepsis (17). Albeit the IL-6 course, with an elevation about 6 h after surgery, corresponds to our data, we found in contrast prolonged elevated IL-6 levels postoperatively with a peak on the first POD, in line with Puchinger et al. (18) However, HA consistently had no significant effect on systemic inflammatory response or clinical outcome, respectively, supporting our results.

### The effect of confounding variables on IL-6 levels

Although present differences between groups, CytoSorb® and control group, including increased ES II, longer surgery duration and CBP times, re-do surgeries, surgeries of the ascending aorta and arch, respectively, a significant impact of these factors on IL-6 levels was only observed for ES II in the confounder analysis. Despite the assumption that patients with an increased ES II will show increased IL-6 levels due to higher morbidity, this was not the case. Interestingly, in those patients with a high ES II of 7 or more and thus suspected high proinflammatory activity, IL-6 levels did not raise above 500 pg/ml. This is explained by the immunologic phenomenon, that critically ill patients are often anergic, characterized by a decrease in cytokine response, described in literature as *compensatory anti-inflammatory response syndrome* (CARS) (18, 19). Putting this into practice, a high ES II renders hemadsorption, with the aim to reduce cytokine levels following CPB, questionable.

### Comparison of IL-6 levels to literature

Moreover, we observed relatively high IL-6 levels with regard to existing literature, with maximum serum concentrations greater than 500 pg/ml, comparable to sepsis patients (20–24). We hypothesized that the significantly elevated IL-6 levels, might be due to the fact that we included not only elective but also acute cardiac surgeries involving complex aortic arch surgeries and circulatory arrest. We did not confirm this hypothesis due to the heterogenous patient population and therefore small number of patients receiving certain operations and due to missing randomization, which is the main limitation of this study. But on another note, as per Schadler et al., the removal of cytokines is described to be concentration-dependent, while low cytokine plasma concentrations show to be not affected, high cytokine plasma levels are ought to be reduced effectively (21). This, although the patients in our cohort exhibited considerably elevated IL-6 levels, was not the case.

### Exploratory analyses

Factors believed to contribute to the inflammatory response following cardiac surgery were included in an exploratory analysis. Literature on differences between oxygenator used in cardiac surgery and postoperative cytokine levels is lacking. We were not able to detect a difference between the three oxygenators used on IL-6 on POD 1. On the contrary, the anti-inflammatory effects of steroids on clinical outcome in cardiac surgery have been investigated in several trials (25–27). The meta-analysis by Dvirnik et al. showed that steroid administration at the time of cardiac surgery had no impact on mortality in over 16,000 patients (27). We analyzed the effect of intraoperative single shot steroid administration [100 mg SOLU-CORTEF® (hydrocortisone sodium succinate)] on IL-6 POD 1 between groups, with no significant difference. CytoSorb® was described to be associated with reduced catecholamine use, which we did not confirm as norepinephrine dosage was comparable in both groups (5.2 ± 4.0 ml/h in the CytoSorb® group vs. 4.8 ± 6.6 in the control group in our study sample) (28, 29). Contrarywise, Santer et al. reported a significantly higher demand for norepinephrine in the HA group in patients undergoing valve surgery for infectious endocarditis. He also found higher reoperation rates due to bleeding going along with a higher need for red blood cell concentrates and platelets with an overall longer hospital stay (30).

We further analyzed other important markers of inflammation including CRP and leucocyte count, which were not influenced by intraoperative HA with CytoSorb®. Also, the ICU stay and overall hospital stay in our cohort was comparable between groups.

Additionally, we were able to confirm the known prognostic effect of ES II, troponin I POD 1 and lactate levels POD 1 on periprocedural mortality in a receiver operating characteristic (ROC) analysis. IL-6 on POD 1 did not show to be of prognostic relevance in our cohort.

Other available data on CytoSorb® in cardiac surgery are smaller retrospective studies and case reports (28, 30–33). In sepsis studies, however, HA already finds an ample field of application (23). In a retrospective septic shock study cohort, the duration of application of CytoSorb® and thus the amount of blood purified seemed to be of clinical importance (23). Asch et al. applied CytoSorb® during CPB, and then continuously for 24 h following cardiac surgery, changing the cartridge every 8 h. However, this had no effect on postoperative inflammatory mediators (12). Gleason et al. combined two Cytosorb cartridges placed in a parallel configuration to reach a total blood flow of about 600 ml/min during CPB (mean duration of CytoSorb® treatment 2.5 ± 1.2 h, range 0.8–5.0 h) to enhance the effect of HA therapy. They reported an initial reduction of the complement factors C3a and C5a, also in the HA group, but this also did not affect outcome (15).

## Limitations

First, one limitation of this study was that the Cytosorb group represented a frailer patient cohort showing significantly higher ES II values. This is partly due to the fact that only the CytoSorb® group included redo surgeries (12 out of 28), a 4.3-fold (11 vs. 3) of ascending aorta replacements, and a 10-fold (10 vs. 1) of aortic arch replacements in comparison to the control group. Hence, significantly longer surgeries and CPB times were found in the CytoSorb® group.

Second this study was not randomized through its retrospective character and a significant negative selection bias might have been introduced by choosing sicker patients for the use of CytoSorb®. On the other side, reduced IL-6 levels were found in patients with an ES II above 7. A reduced general condition is linked to a limited immunologic response in such patients.

Third, in our study we did not find a significant influence of CytoSorb® on mortality. With a mortality of 5.4% (3 out of 56), a sample size of 1,414 patients is required for a logistic regression model with an alpha of 5% (and not 1% as in our study), a power of 95%, a supposed odds ratio of 2.0 and an allocation strategy of 50% for each group. Nevertheless, mortality as endpoint is of use in a meta-analysis and thus just has to be reported also in smaller scale studies.

In future studies we recommend to use ES II for stratification to exclude its effect on immunologic response.

## Conclusion

In conclusion, literature on CytoSorb® in cardiac surgery is diverging and although no clear benefit on the inflammatory response was demonstrated, CytoSorb® is routinely installed in the CPB circuit for the removal of cytokines in complex cardiac surgeries. No significant reduction of postoperative IL-6 levels nor periprocedural mortality through intraoperative HA with CytoSorb®, installed in the CPB in patients undergoing cardiac surgery, was observed. The immunologic response, i.e., IL-6 levels, seems to be reduced in those patients with a high ES II—a poor clinical prognosis, therefore a reduction of IL-6 through HA is not of relevance in these patients. The routine application of CytoSorb® in cardiac surgery to reduce IL-6 needs to be reconsidered. A large multi-institutional trial with stringent entry criteria is required to verify the beneficial impact of hemadsorption in cardiac surgery.

## Data Availability

The raw data supporting the conclusions of this article will be made available by the authors, without undue reservation.
