# Supplementary material for: Impact of CytoSorb® on interleukin-6 in cardiac surgery
Source: Front Cardiovasc Med. 2023 Aug 30;10:1166093. doi: 10.3389/fcvm.2023.1166093 (PMC10498300; doi:10.3389/fcvm.2023.1166093)
Supplement: Supplementary file 1 [file Table1.pdf]

Supplementary Table 1. Interval and ratio scaled baseline parameters of HA and control group. Bold parameters indicate a significant difference between both groups.

		**HA (n=28)**				**Control (n=28)**				**t-Test**		
**time**	**parameter**	**mean**	**sd**	**min**	**max**	**mean**	**sd**	**min**	**max**	**t**	**dof**	**p**
**Preop**	Age	62.8	14.7	32.0	82.0	65.3	10.5	41.0	79.0	0.741	48.80852	0.462
	BMI	27.3	5.4	18.7	45.0	26.8	3.9	20.5	33.5	-0.414	48.79455	0.681
	Euroscore II	5.4	6.0	0.7	23.7	3.0	2.9	0.5	9.8	-1.930	38.66792	0.061
	Heart rate in bpm	73.5	10.1	54.0	90.0	75.5	12.1	53.0	95.0	0.695	52.29134	0.490
	Respiratory rate in bpm	11.8	1.0	9.0	14.0	11.9	0.5	10.0	12.0	0.500	40.70253	0.620
	**Body temperature in °C**	37.1	0.4	36.0	37.7	36.8	0.4	36.0	37.5	-2.224	53.88380	**0.030**
**Baseline**	Hemoglobin (g/dL)	13.3	1.8	11.2	18.0	13.8	1.7	10.5	16.8	0.957	53.97532	0.343
	Leukocyte count (G/L)	7.3	2.2	3.0	12.4	8.0	3.0	3.9	13.3	0.991	50.09947	0.326
	Albumin (g/dL)	42.4	2.4	37.0	48.0	41.0	6.2	29.0	49.0	-1.055	35.02392	0.299
	Bilirubin (mg/dL)	0.5	0.3	0.2	1.4	0.6	0.2	0.3	1.1	0.435	46.33181	0.666
	CRP (mg/L)	9.0	25.3	0.3	135.8	7.3	16.4	0.3	83.2	-0.289	46.15378	0.774
	IL6 (pg/ml)	16.6	39.5	1.5	206.0	8.7	12.7	1.8	67.7	-1.009	32.53396	0.320
	**Procalcitonin (ng/ml)**	0.0	0.0	0.0	0.1	0.1	0.1	0.0	0.2	2.350	30.58273	**0.025**
**Intraop**	Surgery duration (min.)	300.7	72.0	168.0	454.0	291.3	60.0	207.0	422.0	-0.528	52.29416	0.599
	CPB (min.)	155.7	50.2	81.0	253.0	143.3	36.0	77.0	239.0	-1.059	49.00894	0.295
	ACC (min.)	93.3	32.7	0.0	155.0	101.0	36.2	51.0	202.0	0.833	53.46875	0.409

aortic cross clamp – ACC, body mass index – BMI, cardiopulmonary bypass – CPB, C-reactive protein – CRP, Dof - degrees of freedom, hemadsorption – HA, interleukin 6 – IL6, maximum - max, minimum - min, standard deviation – sd, p value - p

Supplementary Table 2. Dichotomous distributed baseline parameters of HA and control group. Bold parameters indicate a significant difference between both groups. Columns 3 and 4 show absolute numbers (n=) and percentages in brackets (%).

		HA (n=28)	Control (n=28)	Chi2 test	
**Time**	**Parameters**			**Chi2**	**p value**
**Preop**	Sex (male)	15 (53.6)	20 (71.4)	1.219	0.270
	Art. Hypertension	21 (75.0)	18 (64.3)	0.338	0.561
	Pulmonary hypertension	2 (7.1)	3 (10.7)	0.220	1.000
	Hyperlipidemia	20 (71.4)	21 (75.0)	0.091	1.000
	COPD	5 (17.9)	4 (14.3)	0.132	1.000
	Diabetes mellitus	7 (21.4)	15 (25)	0.000	1.000
	Peripheral artery disease	0 (0)	0 (0)	NA	NA
	Cerebrovascular disease	7 (25.0)	8 (28.6)	0.091	1.000
	Marfan Syndrome	3 (10.7)	0 (0)	1.409	0.234
**Indication**	Aortic stenosis	7 (25.0)	9 (32.1)	0.088	0.767
	Aortic regurgitation	4 (14.3)	5 (17.9)	0.132	1.000
	Combined aortic vitium	0 (0)	1 (3.6)	0.000	1.000
	Mitral regurgitation	10 (17.9)	12 (28.6)	0.299	0.781
	Mitral stenosis	1 (3.6)	1 (3.6)	0.000	1.000
	Tricuspid regurgitation	4 (21.4)	4 (21.4)	0.000	1.000
	Aneurysm	8 (28.6)	5 (10.7)	0.901	0.543
	Dissection	4 (21.4)	0 (0)	4.308	0.112
	**Coronary artery disease**	9 (32.1)	19 (67.9)	7.142	**0.013**
	Myocardial infarction	3 (0.107)	6 (21.4)	1.191	0.476
**Surgery**	**Re-do surgery**	12 (42.9)	0 (0)	12.833	**0.000**
	Combined surgery	17 (60.7)	17 (46.4)	0.000	1.000
	AVR mechanical	2 (7.1)	0 (ß.0)	2.074	0.492
	AVR biological	8 (28.6)	10 (35.7)	0.082	0.775
	Mitral valve replacement	5 (10.7)	6 (21.4)	0.113	1.000
	Mitral valve repair	1 (3.6)	4 (14.3)	1.977	0.353
	Tricuspid valve repair	2 (7.1)	4 (14.3)	0.187	0.666
	CABG	10 (35.7)	17 (60.7)	3.505	0.112
	Ascending aorta replacement	12 (39.9)	5 (10.7)	4.139	0.073
	**Aortic arch replacement (partial/ full)**	10 (35.7)	1 (3.6)	7.240	**0.007**
	Bentall procedure	1 (3.6)	0 (0.0)	1.018	1.000
	ECMO	1 (3.6)	0 (0.0)	1.018	1.000

Aortic valve replacement – AVR, chronic obstructive pulmonary disease – COPD, coronary artery bypass grafting – CABG, extracorporeal membrane oxygenation – ECMO, Hemadsorption – HA

Supplementary Table 3. Inflammatory blood parameters of both groups specified by median (1st quartile, 3rd quartile). The p-value refers to the Wilcoxon rank sum test.

		HA (n=28)	Control (n=28)	p-value
**IL6 [pg/ml]**	Baseline	3.9 (2.4, 9.8)	3.7 (2.7, 9.8)	0.723
	6h postop	357.5 (261.0, 667.8)	421.5 (191.3, 814.8)	0.919
	POD1	398.5 (215.8, 918.8)	823 (261.8, 1266.0)	0.392
	POD2	203.0 (133.5, 458.5)	314 (85.5, 453)	0.712
**CRP [mg/l]**	Baseline	2.1 (0.9, 6.5)	0.85 (0.3, 5.1)	0.329
	6h postop	14.9 (8.2, 18.7)	14.0 (10.8, 19.1)	0.760
	POD1	67.3 (48.9, 90.8)	77.5 (62.4, 86.6)	0.497
	POD2	232.5 (157.7, 275.4)	241.2 (206.7, 272.8)	0.594
**Leukocyte count (G/l)**	Baseline	7.2 (5.8, 7.9)	6.9 (5.6, 9.4)	0.651
	POD1	11.0 (7.7, 15.1)	2.4 (9.5, 13.5)	0.523
	POD2	12.2 (8.8, 17.2)	14.9 (9.6, 18.5)	0.434

C-reactive protein – CRP, hemadsorption – HA, interleukin 6 – IL6, postoperative – POD

Supplementary Table 4. Postoperative parameters. [a]Pearson's Chi-squared test, [b]Wilcoxon rank sum test with continuity correction, mad … median deviation of the median

	**HA (n=28)**	**Control (n=28)**	**[a]Chi^2/[b]W**	**p-value**
periprocedural mortality, n(%)	3 (10.7)	0 (0)	0.011[a]	0.996
ICU stay, in days median±mad	5.0±2.2	5.0±1.5	378[b]	0.823
atrial fibrillation de novo, n (%)	4 (14.3)	6 (21.4)	0.0004[a]	0.984
stroke rate, n (%)	1 (3.6)	1 (3.6)	0[a]	1.000
ventilation time median±mad	17.5±17.0	19.0±19.3	385[b]	0.914
Reintubation, n (%)	4 (14.3)	0 (0)	0.043[a]	0.836
acute kidney injury, n (%)	3[1] (10.7)	8[1] (28.5)	1.642	0.200
renal replacement therapy, n (%)	0 (0)	0 (0)	0.000[a]	1.000

[1] Patients with **acute kidney injury (AKI) stage I**: Increase in serum creatinine by 0.3mg/dL or more within 48 hours or increase in serum creatinine to 1.5 times baseline according to KDIGO-criteria
